# The search for the ultra‐elusive: Can computed cardiopulmonography enhance early detection of gas exchange abnormality?

**DOI:** 10.1113/EP092925

**Published:** 2025-06-17

**Authors:** Harry B. Rossiter, Yannick Molgat‐Seon

**Affiliations:** ^1^ Institute of Respiratory Medicine and Exercise Physiology The Lundquist Institute for Biomedical Innovation at Harbor–UCLA Medical Center Torrance California USA; ^2^ Department of Kinesiology and Applied Health University of Winnipeg Winnipeg Manitoba Canada; ^3^ Centre for Heart and Lung Innovation The University of British Columbia and St. Paul's Hospital Vancouver British Columbia Canada

**Keywords:** pulmonary circulation, pulmonary function testing, pulmonary gas exchange, V/Q inequality

1

While the mechanisms that control breathing during exercise have been called the ‘ultra‐secret’, the development of clinically useful methods to diagnose abnormalities in pulmonary gas exchange might be called the ‘ultra‐elusive’. Although many methods have been developed over at least a century, most rarely make it out of the physiology laboratory. The lung is so exquisite in its role as a gas exchanger that many potentially devastating pulmonary conditions do not result in overt signs or symptoms, and therefore patients are not investigated with diagnostic testing, until they are advanced to the point that deleterious pulmonary remodelling is irreversible. The clinical utility of developing a simple, rapid, non‐invasive method that is sufficiently sensitive and specific to determine subtle changes in pulmonary gas exchange could therefore make an important impact in earlier diagnosis and treatment of many obstructive, restrictive, fibrotic or pulmonary vascular diseases.

Inert gas washout techniques have been extensively used to better understand heterogeneity of pulmonary deadspace, ventilation and perfusion. In his pioneering work from almost 75 years ago, Ward Fowler (Fowler et al., 1952) described N_2_ clearance characteristics from patients with cardiopulmonary diseases and compared them to those from healthy individuals. The efficiency of gas mixing within the lung was assessed while inhaling 100% O_2_ and assessing the expired N_2_ concentration of subsequent exhalations. During 100% O_2_ breathing, Fowler et al. (1952) found that N_2_ remained detectible in the expirate of patients with asthma, emphysema or pulmonary fibrosis for 7–88 breaths – far greater than the 6–10 breaths observed in young healthy people – indicating unevenness in the ventilatory distribution of the inspired O_2_ within patients’ lungs. The technique, however, relied on cumbersome chemical gas analysis using the Van Slyke technique, and therefore was little appreciated until technological advances, such as automated computing and the respiratory gas mass spectrometer, became more widely available.

Single breath washout, where deadspace and heterogeneity of lung gas distribution are inferred from the profile of N_2_ concentration over the course of a single expirate, and multiple breath washout techniques have seen a resurgence in the 21st century. Multiple breath washout is, for example, superior to plethysmography or spirometry in identifying abnormal lung function in children with cystic fibrosis (Aurora et al., 2005). On the other hand, several other techniques have been less successful in making the leap from laboratory to clinic. Impedance oscillometry or the forced oscillation technique, which involves applying sound waves of different frequencies at the mouth, can determine reactance and resistance within pulmonary airways of different calibers, but evidence of clinical utility is still wanting (Kaminsky et al., 2022). The multiple inert gas elimination technique (MIGET) determines the heterogeneity of alveolar ventilation (${\dot{V}}_{A}$) and perfusion ($\dot{Q}$) and determine the effects on gas exchange of ${\dot{V}}_{A}/\dot{Q}$ inequality, shunt and diffusion limitation. MIGET is perhaps the gold‐standard of gas exchange measurements and has been used in extensive physiological investigations of gas exchange at rest and during exercise (Hopkins, 2020). However, the complexity of application and the invasive nature of MIGET has hindered its clinical translation. Several imaging techniques, such as single‐photon emission computed tomography, positron emission tomography, magnetic resonance imaging, computed tomography and electrical impedance tomography, or use of fluorescent and radiolabelled microspheres, each come with their own barriers to implementation including factors of cost, time or radiation exposure (Hopkins, 2020).

The new kid on the block is Peter Robbins' computed cardiopulmonography (CCP) approach (Alamoudi et al., 2026; Mountain et al., 2018). The CCP method aims to quantify pulmonary function inhomogeneity that contributes to ${\dot{V}}_{A}/\dot{Q}$ inequalities, using a 15‐min multiple breath washout test. CCP has strong potential for clinical application due to its relative simplicity, non‐invasive and radiation‐free nature. While CCP relies on many of the same concepts as Fowler's original 1952 approach, it uses technologically advanced in‐line molecular flow sensing by laser absorption spectroscopy, to provide O_2_, CO_2_ and water vapor concentration data of sufficient fidelity (every 10 ms), and under the same temperature and pressure conditions as the measurement of gas flow, to recover parameters reflecting pulmonary deadspace volume, functional residual capacity and inhomogeneity. Outputs from this technique are then compared to computational models of a typical, or reference, lung to resolve parameters reflecting inhomogeneity in deadspace, compliance and vascular conductance (Mountain et al., 2018). The approach is sensitive to inhomogeneity among, for example, healthy young and older adults or individuals with obstructive pulmonary disease (Mountain et al., 2018) or cystic fibrosis.

To translate CCP into clinical use first requires establishment of normal values. In this issue of *Experimental Physiology* Alamoudi et al. (2026) take a first step towards developing reference ranges for novel CCP variables. The influence of physical characteristics – age, sex and height – on pulmonary function, renders the interpretation of clinically meaningful diagnostic thresholds relatively complex. In their article, Alamoudi et al. (2026) highlight that, of the parameters derived from CCP, those that are ‘volume‐related’ (functional residual capacity and deadspace volume) were affected by age and height, whereas those that are ‘inhomogeneity‐related’ (standardised deadspace and the standard deviation for the natural logarithm of the standardised lung compliance) were only affected by age. Thus, inhomogeneity‐related CCP parameters may be particularly useful for the early detection of pulmonary gas exchange abnormalities given their insensitivity to physical characteristics that can vary across the lifespan. From an operational standpoint, these measures may be appealing since, unlike current spirometric or plethysmographic assessments, they would only need to be interpreted in the context of the age‐related decline in pulmonary function.

Like any new technique, CCP must undergo rigorous investigation, testing and validation before it can be extended to clinical settings, which provides exciting opportunities for research. While the study of Alamoudi et al. (2026) provides an important step in the direction of wider clinical application for CCP, their reference group is incompletely characterized in terms of the normalcy of pulmonary function and gas exchange. Given the importance of age on inhomogeneity‐related CCP parameters, a comprehensive assessment of pulmonary function, chest imaging and arterial blood gases from a large normal, multi‐ethnic cohort of all ages will be needed to develop robust reference values for CCP. Also, in light of the wide prevalence of obesity, a more comprehensive investigation of the influence of body habitus, particularly abdominal adiposity, on CCP variables would also be of great interest. An understanding of therapeutic interventions, environmental factors, or physiological stressors such as exercise on CCP parameters would help add construct validity for the technique. Indeed, the development of CCP, and its potential clinical applicability, will likely inspire a great deal of additional research to address these important questions as well as other fundamental aspects of CCP before it can enter the clinical proving ground.

Scientific discovery is hard fought and tortuous. We marvel at Fowler's original insight to develop the concepts of pulmonary clearance ∼75 years ago and watch, with bated breath, the evolution of a new technique as it attempts to shine new light on the ‘ultra‐elusive’ problem of early detection of pulmonary gas exchange abnormalities.

## AUTHOR CONTRIBUTIONS

All authors have read and approved the final version of this manuscript and agree to be accountable for all aspects of the work in ensuring that questions related to the accuracy or integrity of any part of the work are appropriately investigated and resolved. All persons designated as authors qualify for authorship, and all those who qualify for authorship are listed.

## CONFLICT OF INTEREST

H.R. reports consulting fees from the NIH RECOVER‐ENERGIZE working group (1OT2HL156812), and is involved in contracted clinical research with GlaxoSmithKline, Genentech, Intervene Immune, Mezzion, Regeneron, Respira, Roche and United Therapeutics. He is a visiting Professor at the University of Leeds, UK and the University of Pavia, Italy. He reports a pending patent application filed by The Lundquist Institute, titled ‘Testing System to Diagnose Neuromuscular Deconditioning and Pathologic Conditions’.
